# Amyotrophic lateral sclerosis with *SOD1* mutations shows distinct brain metabolic changes

**DOI:** 10.1007/s00259-021-05668-7

**Published:** 2022-01-25

**Authors:** Antonio Canosa, Andrea Calvo, Cristina Moglia, Rosario Vasta, Francesca Palumbo, Luca Solero, Francesca Di Pede, Sara Cabras, Vincenzo Arena, Grazia Zocco, Federico Casale, Maura Brunetti, Luca Sbaiz, Salvatore Gallone, Maurizio Grassano, Umberto Manera, Marco Pagani, Adriano Chiò

**Affiliations:** 1 ALS Centre, ‘Rita Levi Montalcini’ Department of Neuroscience, University of Turin, Turin, Italy; 2 Azienda Ospedaliero-Universitaria Città della Salute e della Scienza di Torino, SC Neurologia 1U, Turin, Italy; 3 Institute of Cognitive Sciences and Technologies, C.N.R., Rome, Italy; 4 Neuroscience Institute of Turin (NIT), Turin, Italy; 5 Positron Emission Tomography Centre AFFIDEA-IRMET S.p.A., Turin, Italy; 6 Laboratory of Genetics, Department of Clinical Pathology, Azienda Ospedaliero-Universitaria Città della Salute e della Scienza di Torino, Turin, Italy; 7 Department of Medical Radiation Physics and Nuclear Medicine, Karolinska University Hospital, Stockholm, Sweden

**Keywords:** Amyotrophic lateral sclerosis (ALS), *SOD1*, ^18^F-FDG-PET, Brain metabolism

## Abstract

**Purpose:**

Neuropathological data suggest that ALS with *SOD1* mutations (*SOD1*-ALS) is a distinct form of ALS. We evaluated brain metabolic changes characterizing *SOD1*-ALS as compared to sporadic ALS (sALS), employing ^18^fluorodeoxyglucose-positron-emission tomography (^18^F-FDG-PET).

**Methods:**

We included 18 *SOD1*-ALS patients, 40 healthy controls (HC), and 46 sALS patients without mutations in *SOD1*, *TARDBP*, *FUS*, and *C9ORF72*, randomly selected from 665 subjects who underwent brain ^18^F-FDG-PET at diagnosis between 2008 and 2019 at the ALS Centre of Turin. We excluded patients with frontotemporal dementia. We used the full factorial design in SPM12 to evaluate whether differences among groups exist overall. In case the hypothesis was confirmed, group comparisons were performed through the two-sample *t*-test model of SPM12. In all the analyses, the height threshold was *P* < 0.001 (*P* < 0.05 FWE-corrected at cluster level).

**Results:**

The full factorial design resulted in a significant main effect of groups. We identified a relative hypometabolism in sALS patients compared to *SOD1*-ALS cases in the right precentral and medial frontal gyrus, right paracentral lobule, and bilateral postcentral gyrus. *SOD1* patients showed a relative hypermetabolism as compared to HC in the right precentral gyrus and paracentral lobule. As compared to HC, sALS patients showed relative hypometabolism in frontal, temporal, and occipital cortices.

**Conclusion:**

S*OD1*-ALS was characterized by a relative hypermetabolism in the motor cortex as compared to sALS and HC. Since promising, targeted, therapeutic strategies are upcoming for *SOD1*-ALS, our data support the use of PET to study disease pathogenesis and to track its course in clinical trials, in both asymptomatic and symptomatic mutation carriers.

**Supplementary Information:**

The online version contains supplementary material available at 10.1007/s00259-021-05668-7.

## Introduction

Amyotrophic lateral sclerosis is characterized by the progressive degeneration of upper and lower motor neurons, leading to voluntary muscle wasting and weakness, and causing death within 2–5 years from onset [1]. Approximately 10% of cases are familial (fALS), while 90% are thought to be sporadic (sALS) [2]. *SOD1* mutations were identified as a cause of fALS in 1993 [3]. Afterwards, they were detected also in ~1% of sALS cases [2]. *SOD1-*ALS seems to be a distinct form of ALS, since the neuropathological hallmark in ~95% of ALS cases is the cytoplasmic mislocalization and aggregation of hyperphosphorylated TDP-43 (pTDP-43) within neurons and glia, while in *SOD1*-ALS, no pTDP43 staining is detected in the motor cortex [4]. The study of the peculiarities of the neurodegenerative cascade in *SOD1*-ALS has leaded to the promising development of an antisense oligonucleotide that mediates the degradation of SOD1 messenger RNA to reduce SOD1 protein synthesis [5]. Brain ^18^fluorodeoxyglucose-positron-emission tomography (^18^F-FDG-PET) is a powerful tool to investigate the involvement of brain structures and functions in ALS in vivo [6], and is a potentially useful biomarker for the assessment of the extent of brain lesions [7]. Recent works have demonstrated that ^18^F-FDG-PET is able to disclose the metabolic brain correlates of different aspects of ALS phenotype, including the extent of motor deficits [8], and the presence of cognitive [9] and behavioral impairment [10]. A number of studies [11, 12] highlighted the metabolic features which characterize patients carrying the *C9ORF72* expansion. Otherwise, ^18^F-FDG-PET investigations focused on *SOD1* mutation carriers are still lacking. Therefore, the aim of the present study was the identification of brain metabolic changes characterizing *SOD1*-ALS as compared to sALS, employing ^18^F-FDG-PET.

## Materials and methods

### Study participants

We included 18 patients carrying *SOD1* mutations, diagnosed with genetically determined ALS according to El Escorial revised diagnostic criteria [13] at the ALS Centre of Turin (‘Rita Levi Montalcini’ Department of Neuroscience, University of Turin, Turin, Italy), between 2008 and 2019. A comparison cohort of patients diagnosed with definite, probable, and probable laboratory-supported sALS according to El Escorial revised diagnostic criteria [13] was randomly collected from the series of 665 subjects who underwent brain ^18^F-FDG-PET at diagnosis in the same time period at the ALS Centre of Turin. The sALS cohort included subjects without mutations in the major ALS-related genes (i.e. *SOD1*, *TARDBP*, *FUS*, *C9ORF72*). Fifty sALS were originally collected. Then, they were reduced to 46 because we excluded 4 patients with frontotemporal dementia (FTD), since no patients in our *SOD1* group had FTD, in agreement with published literature suggesting that dementia is rare in patients carrying mutations of this gene [14, 15]. Since *SOD1* patients more often show a flail-leg phenotype with prevalent lower motor neuron involvement as compared to non-carriers [16, 17], we evaluated patients’ motor phenotype in order to explain eventual brain metabolic differences. We applied a modified version of the classification proposed by Pioro and colleagues [17] for phenotypic classification: (1) ALS, if lower motor neuron (LMN) signs (i.e., fasciculations, atrophy, and weakness) and unequivocal upper motor neuron (UMN) signs (i.e., spasticity, Babinski sign, Hoffmann sign, and clonus) were present; (2) ALS with probable UMN signs (ALS-PUMNS), in the presence of prominent LMN signs with preserved or mild to moderately hyperactive stretch reflexes but without spasticity, extensor plantar responses, Hoffmann sign, or clonus; and (3) pure LMN, where only LMN signs were evident. Furthermore, the King’s stage at PET was obtained for sALS and *SOD1* patients from the ALSFRS-R scale fulfilled at the time of PET scan, according to a published algorithm [18].

We included in the analyses 40 healthy controls (HC). We considered eligible as controls subjects referred to the PET Centre for suspected lung cancer (i) with no oncologic disease detected, (ii) with brain PET scan reported as normal by the nuclear medicine physician, (iii) without history of neurological disorders, and (iiii) with normal neurological examination.

### Genetic analysis

All patients underwent genetic analysis for *C9ORF72*, *SOD1*, *TARDBP*, and *FUS* genes. All the coding exons and 50 bp of the flanking intron-exon boundaries of *SOD1*, of exon 6 of *TARDBP*, and of exons 14 and 15 of *FUS* have been PCR amplified, sequenced using the BigDye Terminator v3.1 sequencing kit (Applied Biosystems Inc.), and run on an ABIPrism 3500 genetic analyzer. These exons were selected as the vast majority of known pathogenic variants are known to lie within these mutational hotspots. A repeat-primed PCR assay was used to screen for the presence of the GGGGCC hexanucleotide expansion in the first intron of *C9ORF72.* A cut-off of ≥30 repeats was considered pathological [19].

### ^*18*^F-FDG-PET acquisition

Brain ^18^F-FDG-PET was performed according to published guidelines [20]. Patients fasted at least 6 h before the exam. Blood glucose was <7.2 mmol/l in all cases before the procedure. After a 20-min rest, about 185 MBq of ^18^F-FDG was injected. The acquisition started 60 min after the injection. In the patient group, a whole-body scan was performed setting head-first. In the control group, a separate brain scan was performed after the whole-body one with a time difference of 15 min. The ^18^F-FDG-PET acquisition procedure was performed in the same environmental conditions in patients and controls, according to published guidelines [20]. PET/CT scans were performed on a Discovery ST-E System (General Electric). Brain CT (slice thickness of 3.75 mm, 140 kV, 60–80 mAs), and PET scan was sequentially acquired, the former being used for attenuation correction of PET data. The PET images were reconstructed with 4 iterations and 28 subsets with an initial voxel size of 2.34 × 2.34 × 2.00 mm, and data were collected in 128 × 128 matrices.

### Statistical analysis

The demographic and clinical characteristics of patient groups (*SOD1* patients and sALS patients) and HC were compared as follows. The *χ*^2^-test was employed for categorical variables. The Mann-Whitney test was used for quantitative, continuous variables.

SPM12 implemented in Matlab R2018b (MathWorks, Natick, MA, USA) was used for image spatial normalization to a customized brain ^18^F-FDG-PET template [21]. Normalization with a subcortical reference region was not considered as an option, since all brain regions have been demonstrated to be potentially affected in ALS. Intensity normalization was performed using the 0.8 default SPM value of grey matter threshold, and images were smoothed with a 10-mm filter and submitted to statistical analysis.

Although the scope of the study was the assessment of metabolic differences between sALS and *SOD1*-related ALS, for a more exhaustive characterization of patient metabolic state, we also included HC in the analyses.

We used the full factorial design as implemented in SPM12 to test the hypothesis that differences among groups (*SOD1*, sALS, HC) exist overall (i.e., main effect of groups). Age at PET and sex were used as covariates, and the height threshold was set at *P* < 0.001 (*P* < 0.05 FWE-corrected at cluster level). In case the hypothesis was confirmed, comparisons among groups were performed through the two-sample *t*-test model of SPM12.

We compared *SOD1* and sALS patients including age at PET, sex, site of onset (spinal/bulbar), and King’s stage at PET as covariates. King’s stage was included as covariate since it influences brain metabolism [8]. In the comparison of each patient group with HC, age at PET and sex were used as covariates. In all group comparisons, the height threshold was set at *P* < 0.001 (*P* < 0.05 FWE-corrected at cluster level). In all the analyses, only clusters containing >125 contiguous voxels were considered significant. Brodmann areas (BAs) were identified at a 0–2-mm range from the Talairach coordinates of the SPM output isocentres corrected by Talairach Client (http://www.talairach.org/index.html).

## Results

### Demographic, clinical, and genetic data

The comparison of demographic and clinical data of *SOD1*-patients and sALS cases is reported in Table 1. We found significant differences between *SOD1*-patients and sALS subjects for site of onset and motor phenotype. Bulbar onset was less frequent in the *SOD1* group, in agreement with population-based data from Italy [16]; the pure LMN phenotype was present only in the *SOD1*-ALS, in agreement with the published literature reporting that such phenotypic variant is frequent among *SOD1* mutations carriers [15, 16]. The median age at PET of HC (66.5 years, IQR 57.1–69.0) did not result significantly different from *SOD1* (*P* = 0.346) and sALS patients (*P* = 0.372). In the control group, 29 subjects were male (72.5%) and 11 female (27.5%). Sex distribution resulted different as compared to *SOD1* (*P* = 0.015) and sALS subjects (*P* = 0.004). The effect of site of onset and sex on the results was kept under control including them as covariates in the analyses.

**Table 1:** Comparison of demographic and clinical characteristics of sALS and *SOD1*-ALS patients. Significant differences are reported in bold. ALSFRS-R: ALS Functional Rating Scale – Revised. Pure LMN: pure lower motor neuron phenotype. ALS-PUMNS: ALS with probable Upper Motor Neuron signs

	sALS *N* = 46	*SOD1*-ALS *N* = 18	
	*Median (IQR)*	*Median (IQR)*	*p*
Age at PET (years)	65.4 (58.2–74.5)	62.2 (57.1–69.0)	*p* = 0.110
ALSFRS-R total score	40.0 (33.7–43.0)	38.0 (35.7–40.2)	*p* = 0.811
	*n (%)*	*n (%)*	
Sex			*p* = 0.860
*Female*	27 (58.7)	11 (61.1)	
*Male*	19 (41.3)	7 (38.9)	
Site of onset			***p*** **=** **0.006**
*Bulbar onset*	19 (41.3)	1 (5.6)	
*Spinal onset*	27 (58.7)	17 (94.4)	
Motor phenotype			***p*** **=** **0.003**
*Pure LMN*	0 (0.0)	4 (22.2)	
*ALS-PUMNS*	24 (52.2)	5 (27.8)	
*ALS*	22 (47.8)	9 (50.0)	
King’s staging			
*Stage 1*	13 (28.3)	6 (33.3)	*p* = 0.711
*Stage 2*	14 (30.4)	5 (27.8)	
*Stage 3*	16 (34.8)	7 (38.9)	
*Stage 4a/4b*	3 (6.5)	0 (0.0)	

Among *SOD1* patients, four carried the p.Gly94Asp (c.281G > A) mutation, three the p.Leu145Phe (c.435G > C), and two the p. Asp110Tyr (c.328G > T). The following 9 mutations were found in one patient each: p.Gly148Cys (c.442G > T), p.Gly42Ser (c.124G > A), p.Leu85Phe (c.255G > C), p.Asn66Thr (c.197A > T), p.Ser108LeufsTer15 (c.320_321insT), p.Gly73Ser (c.217G > A), p.Asn20Ser (c.59A > G), p.Val6Met (c.16G > A), and p.Asn66Ser (c.197A > G).

### ^*18*^F-FDG-PET data

#### Full factorial analysis

The full factorial design resulted in a significant main effect of groups (Supplemental Figure 1) in clusters mainly including frontal and occipital regions. We hence computed the post hoc comparisons among the three groups.

#### SOD1-ALS vs sALS

**Fig. 1:**
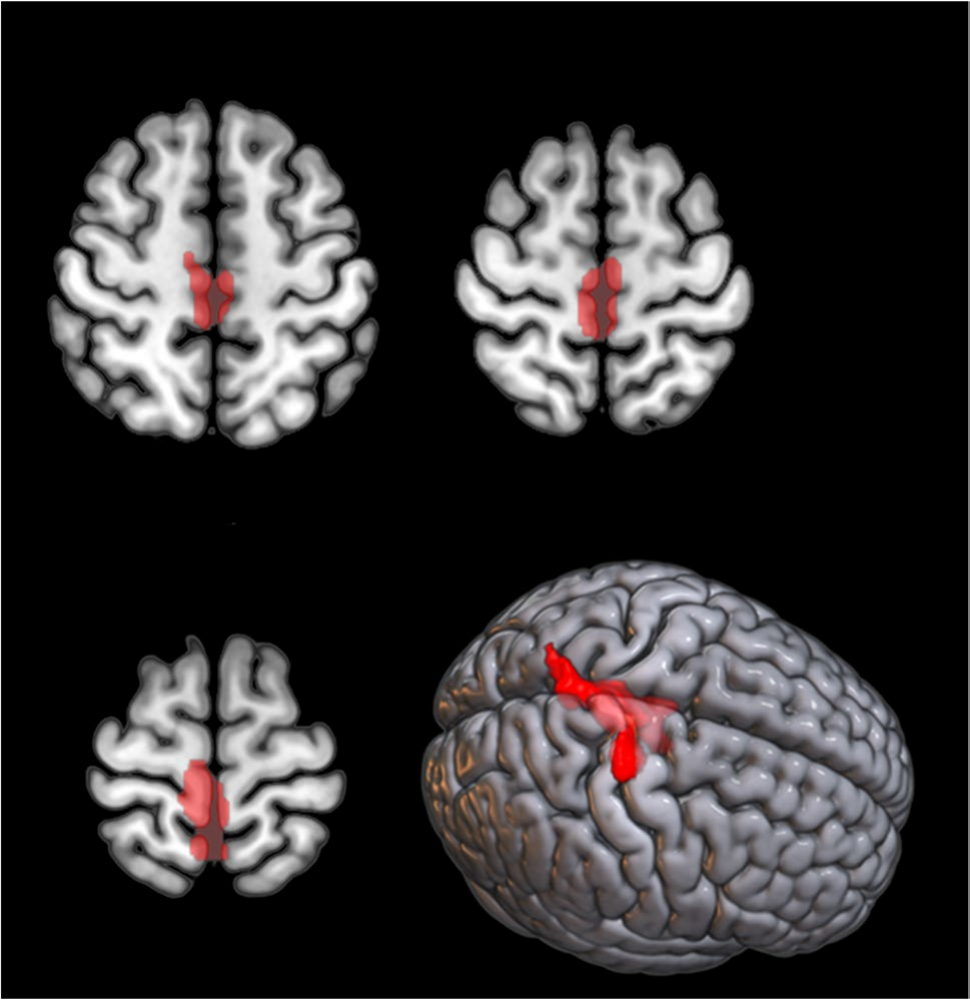
*SOD1* group *versus* sALS group. The regions showing a statistically significant relative hypometabolism in the sALS group as compared to the *SOD1* group are marked in red and are reported on axial sections of a brain magnetic resonance imaging template and on the brain surface of a glass brain rendering (bottom right)

We identified a relative hypometabolism in the sALS group compared to the *SOD1* group in a cluster including the right precentral and medial frontal gyrus, the right paracentral lobule, and bilateral postcentral gyrus (Figure 1; Table 2). We did not identify any cluster of relative hypermetabolism in sALS patients as compared to *SOD1* patients.

**Table 2:** Clusters showing a statistically significant relative hypometabolism in the sALS group as compared to the *SOD1* group (BA = Brodmann area)

p(FWE-corrected)	Cluster extent	*Z*-score	Talairach coordinates			Lobe	Cortical region	BA
0.000	1473	4.09	4	−34	68	Frontal	Right paracentral lobule	4
		3.74	32	−22	71	Frontal	Right precentral gyrus	4
		3.69	−4	−53	71	Parietal	Left postcentral gyrus	7
		3.54	2	−49	69	Parietal	Right postcentral gyrus	7
		3.47	8	−15	50	Frontal	Right medial frontal gyrus	6

#### SOD1-ALS vs HC

**Fig. 2:**
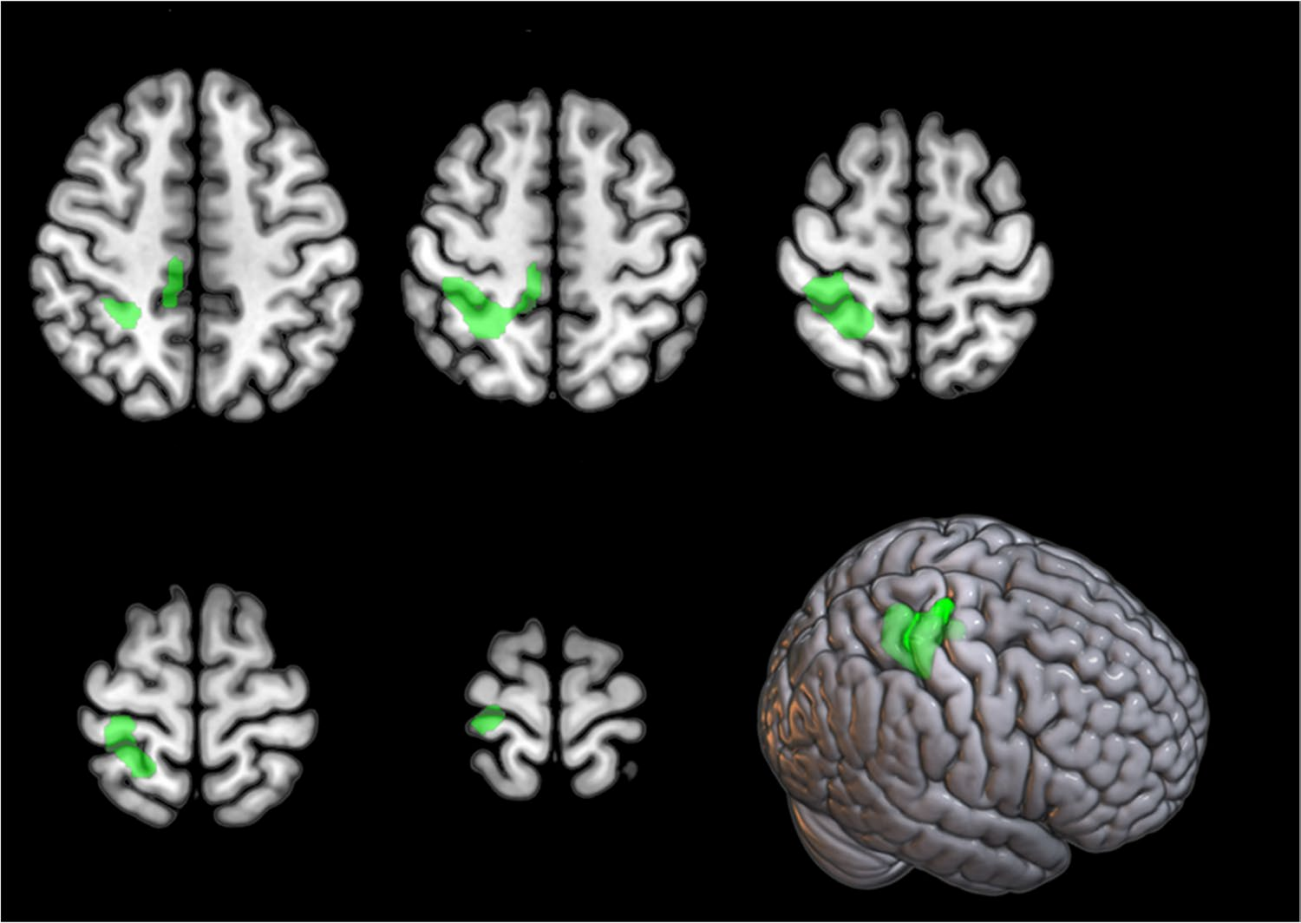
*SOD1* group *versus* HC. The regions showing a statistically significant relative hypermetabolism in *SOD1* patients as compared to HC are marked in green and are reported on axial sections of a brain magnetic resonance imaging template and on the brain surface of a glass brain rendering (bottom right)

We did not find any cluster of relative hypometabolism in the *SOD1* group as compared to HC. *SOD1* patients showed a cluster of relative hypermetabolism as compared to HC, including the right precentral gyrus and paracentral lobule (Figure 2; Table 3).

**Table 3:** Clusters showing a statistically significant relative hypermetabolism in the *SOD1* group as compared to healthy controls (BA = Brodmann area)

p(FWE-corrected)	Cluster extent	*Z*-score	Talairach coordinates			Lobe	Cortical region	BA
0.000	1094	4.74	26	−42	56	Parietal	Right sub-gyral gray matter	40
		3.84	26	−24	69	Frontal	Right precentral gyrus	4
		3.70	8	−25	49	Frontal	Right paracentral lobule	6
		3.68	10	−32	50	Frontal	Right paracentral lobule	5

#### sALS vs HC

**Fig. 3:**
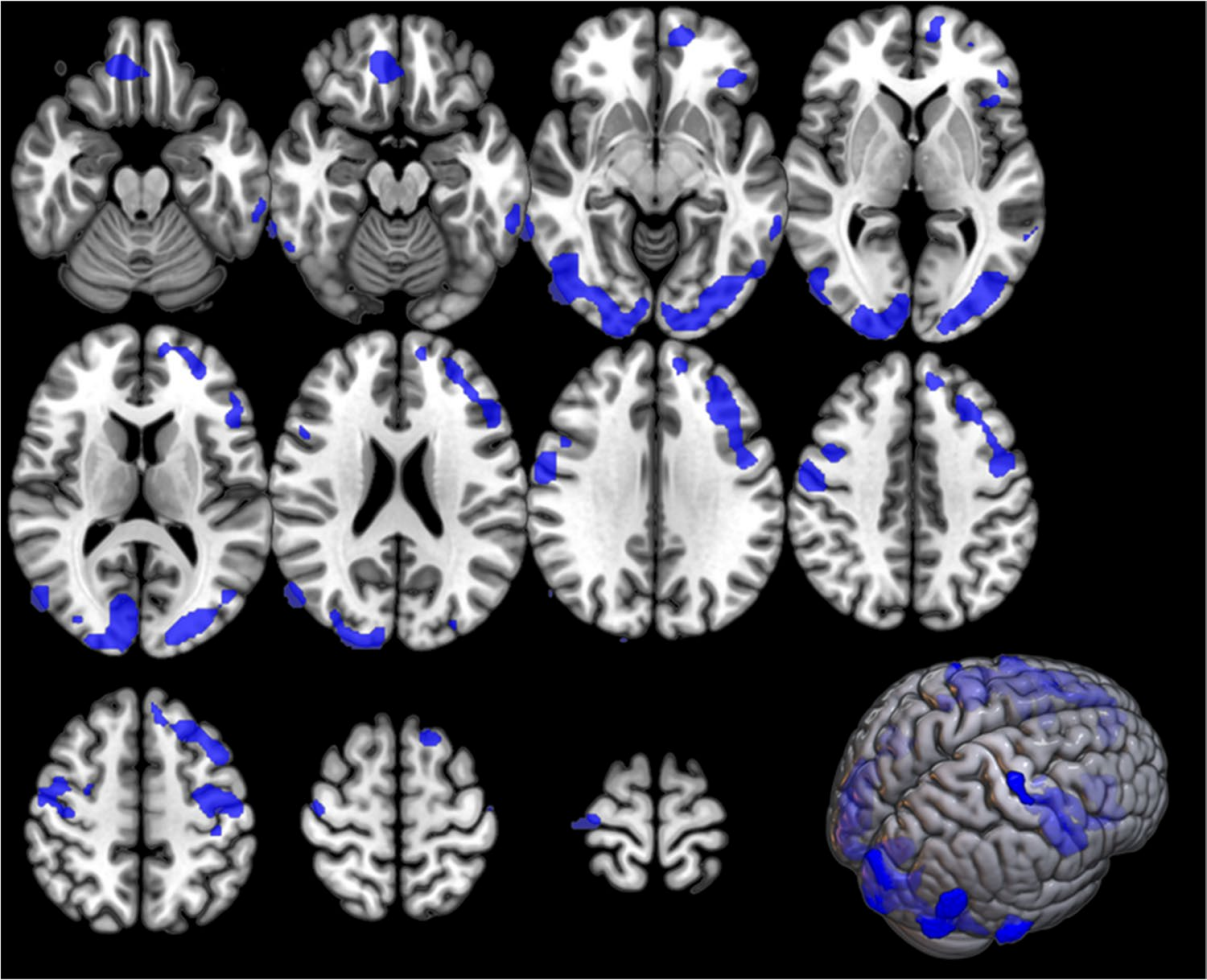
sALS group *versus* HC. The regions showing a statistically significant relative hypometabolism in sALS patients as compared to HC are marked in blue and are reported on axial sections of a brain magnetic resonance imaging template and on the brain surface of a glass brain rendering (bottom right)

As compared to HC, sALS patients showed large clusters of relative hypometabolism, mainly in frontal, temporal, and occipital cortices (Figure 3; Table 4). We did not find any cluster of relative hypermetabolism in sALS patients as compared to HC.

**Table 4:** Clusters showing a statistically significant relative hypometabolism in the sALS group as compared to HC (BA = Brodmann area)

p(FWE-corrected)	Cluster extent	*Z*-score	Talairach coordinates			Lobe	Cortical region	BA
0.001	1148	4.59	55	−6	37	Frontal	Right precentral gyrus	6
		3.87	42	6	37	Frontal	Right precentral gyrus	9
		3.61	40	−15	50	Frontal	Right precentral gyrus	4
		3.55	42	−1	50	Frontal	Right middle frontal gyrus	6
		3.48	48	13	23	Frontal	Right inferior frontal gyrus	9
		3.29	44	−20	65	Parietal	Right postcentral gyrus	3
0.000	5775	4.48	−36	−76	−5	Occipital	Left inferior occipital gyrus	19
		4.47	69	−45	−8	Temporal	Right middle temporal gyrus	21
		4.31	−42	−74	6	Occipital	Left middle occipital gyrus	19
		4.13	53	−71	16	Temporal	Right middle temporal gyrus	39
		3.95	−61	−45	−10	Temporal	Left middle temporal gyrus	21
		3.95	10	−89	15	Occipital	Right cuneus	18
		3.90	51	−76	−1	Occipital	Right middle occipital gyrus	19
		3.89	24	−95	8	Occipital	Right middle occipital gyrus	18
		3.80	−24	−93	1	Occipital	Left cuneus	18
		3.79	24	−92	23	Occipital	Right cuneus	19
		3.76	50	−64	−4	Temporal	Right inferior temporal gyrus	19
		3.61	32	−80	−4	Occipital	Right inferior occipital gyrus	18
		3.56	6	−86	−1	Occipital	Right lingual gyrus	18
0.000	3752	4.24	−50	2	37	Frontal	Left precentral gyrus	6
		4.20	−12	54	−6	Frontal	Left superior frontal gyrus	10
		4.19	−40	16	49	Frontal	Left superior frontal gyrus	8
		4.09	−50	26	19	Frontal	Left inferior frontal gyrus	45
		4.04	−32	29	32	Frontal	Left middle frontal gyrus	9
		4.04	−36	36	26	Frontal	Left superior frontal gyrus	9
		3.92	−38	31	−10	Frontal	Left inferior frontal gyrus	47
		3.86	−30	49	10	Frontal	Left middle frontal gyrus	10
		3.82	−28	27	43	Frontal	Left middle frontal gyrus	8
		3.71	−50	−11	50	Parietal	Left postcentral gyrus	3
		3.55	−16	26	54	Frontal	Left superior frontal gyrus	6
0.029	521	4.19	18	35	−30	Frontal	Right orbital gyrus	11
		4.03	8	38	−17	Frontal	Right medial frontal gyrus	11

## Discussion

In the present study, we evaluated the brain metabolic hallmarks of *SOD1*-ALS as compared to sporadic ALS. In the direct comparison between *SOD1* and sALS patients, sALS subjects showed a relative hypometabolism in motor regions. Strikingly, in the comparison of each group with HC, the same motor areas resulted to be relatively hypometabolic in the sALS group and relatively hypermetabolic in the *SOD1* group.

The clusters of relative hypometabolism of sALS patients as compared to HC were mainly located in the frontal and occipital cortex, in agreement with the results of a previous study comparing ALS patients and healthy controls [22].

The finding of a relative hypometabolism in the motor cortex in sALS patients as compared to *SOD1* subjects might be related to the phenotypic differences between the two groups. Indeed, upper motor neuron signs were detected more frequently in the sALS group, while *SOD1* patients showed a prevalence of lower motor neuron signs in half of cases. This phenotypic heterogeneity between the two groups is in line with published population-based studies reporting that *SOD1*-ALS is usually associated with a prevalent lower motor neuron, flail leg picture [15, 16]. Indeed, neuropathological studies report that motor cortex burden is less severe in *SOD1*-ALS than that observed in sporadic patients with TDP-43 proteinopathy [4]. A recent MRI study did not identify any difference in cortical thickness between *SOD1*-ALS patients and sALS subjects [23]. Nevertheless, cortical metabolic changes detected by ^18^F-FDG-PET may precede gray matter atrophy identified by structural MRI, and therefore may be identified earlier along the disease course [24]. Indeed, a PET study comparing sALS cases and patients who were homozygous for the p.D91A *SOD1* mutation showed a relative reduction in cortical ^11^C-flumazenil binding in the motor and motor association regions of sALS patients, possibly due to the loss of neurons bearing the GABA-A receptors [25]. In order to evaluate whether the metabolic differences between *SOD1* and sALS patients were entirely explainable based on the different clinical involvement of upper motor neuron, we repeated the analysis including the motor phenotype among covariates. The results were unchanged (data not shown). Such findings suggest that the metabolic changes that we still observe are due to a different involvement of motor cortical regions between the two groups, that goes beyond the different clinical phenotype. The interpretation of the relative hypermetabolism of the motor cortex in *SOD1* cases as compared to HC is more challenging. A recent PET study including asymptomatic *SOD1* mutations carriers and *SOD1*-ALS patients reported an increased uptake of a tracer of microglia activation (^11^C-PK11195) in the motor cortex in symptomatic subjects [26]. ^18^F-FDG-PET was not performed in that study, but the authors hypothesized that microglial activation, together with astrocyte reaction, might be a phenomenon underlying the finding of glucose hypermetabolism, that has been extensively reported in ALS. This hypothesis is in line with data based on animal models of cerebral ischemia, showing that microglial activation, evidenced by an increased ^11^C-PK11195 uptake, is associated with increased ^18^F-FDG uptake [27]. Otherwise, brain double tracer PET studies employing both ^11^C-PK11195 and ^18^F-FDG in mild cognitive impairment and early onset Alzheimer’s disease showed microglial activation overlapping clusters of hypometabolism, suggesting a detrimental contribution of microglia in neuron degeneration [28, 29]. A recent study including subjects with mild cognitive impairment showed that higher tau-PET uptake was associated with higher glucose metabolism in patients with lower levels of amyloid-PET uptake. This finding resulted to be associated with lower memory performance [30]. A study focused on the identification of a brain ^18^F-FDG-PET Parkinson’s disease–related pattern (PDRP) showed that the typical PDRP topography was characterized by relative hypermetabolism in the thalamus, putamen/pallidum, pons, cerebellum, and motor cortex. These changes co-varied with relatively decreased metabolism in the prefrontal, parietal, temporal, and occipital cortices. The topography of metabolic alterations was interpreted as reflecting changes in corticostriatopallido-thalamocortical circuits and related pathways in Parkinson’s disease [31]. ^18^F-FDG-PET hypermetabolism was reported also in Prion disease, situated in many subcortical regions, especially those in limbic and mesolimbic systems. Such findings were interpreted as possibly related to microglial activation, since there is some evidence for microglial activation in Creutzfeldt-Jakob disease [32]. Compiling these data into a coherent framework is challenging. Indeed, the role of microglia in ALS can be ambivalent. Data from the *SOD1* transgenic mouse suggest that it can be protective in the early phases and detrimental with the progression of the disease [33]. The importance of its involvement is further supported by studies based on genetic manipulation of mouse models and showing that mutant SOD1 must be expressed in microglia and astrocytes, in addition to motor neurons, to produce the ALS-like phenotype [34].

Our study has some limitations. First, the relatively small size of the *SOD1* group might have influenced our findings. Nevertheless, almost all published neuroimaging studies including *SOD1*-ALS patients have a smaller sample size than the present study, and none of them employed brain ^18^F-FDG-PET [35]. Second, we included patients carrying different *SOD1* mutations. Nevertheless, neuropathological data point to *SOD1*-ALS as a relatively homogenous entity [4]. Third, we could not perform partial volume effect correction for cortical atrophy, since brain MRI was not available for all patients. However, according to previous studies, including voxel-based atrophy correction of resting glucose metabolism, metabolic measurement resulted to be relatively independent of cortical atrophy [36].

The recent European Academy of Nuclear Medicine and European Academy of Neurology recommendations about the use of brain ^18^F-FDG-PET did not suggest its clinical use in ALS. Accordingly, our data support its application in a research setting, including clinical trials [37]. We found that *SOD1*-ALS was characterized by a relatively higher metabolism in the motor cortex as compared to sporadic ALS and healthy controls. Since promising, targeted, therapeutic strategies are upcoming for *SOD1*-ALS [5], the study of the neurodegenerative process associated with this genetic form of ALS through PET imaging can provide potentially useful biomarkers to understand disease pathogenesis and to track the disease course in clinical trials, in both asymptomatic and symptomatic mutation carriers.

## Supplementary information

Supplemental Fig. 1. Full factorial analysis including *SOD1* patients, sALS patients, and healthy controls. The regions showing a significant main effect of groups are marked in yellow and are reported on axial sections of a brain Magnetic Resonance Imaging template and on the brain surface of a glass brain rendering (bottom right) (PNG 1062 kb)

High resolution (TIF 372 kb)
