# Supplementary figures and images for: Amyotrophic lateral sclerosis with SOD1 mutations shows distinct brain metabolic changes
Source: Eur J Nucl Med Mol Imaging. 2022 Jan 25;49(7):2242–50. doi: 10.1007/s00259-021-05668-7 (PMC9165265; doi:10.1007/s00259-021-05668-7)

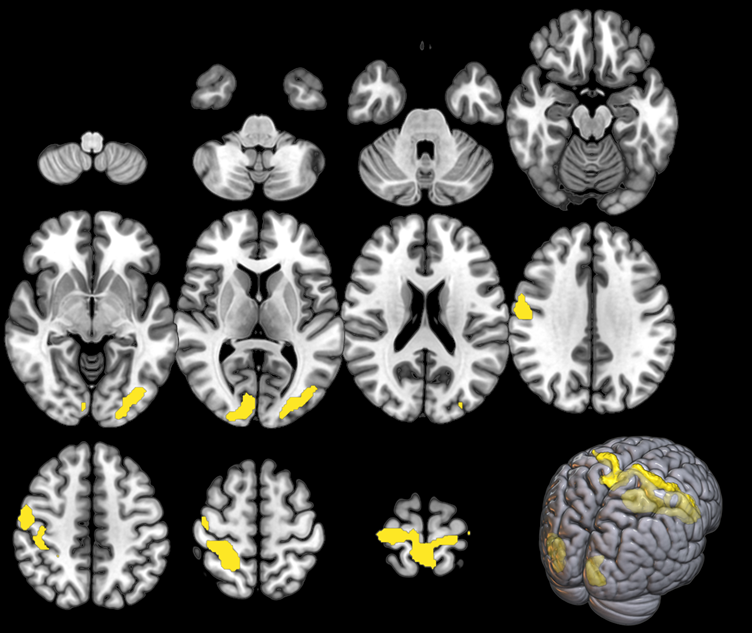

Supplement: Supplementary file 1 — Full factorial analysis including SOD1 patients, sALS patients, and healthy controls. The regions showing a significant main effect of groups are marked in yellow and are reported on axial sections of a brain Magnetic Resonance Imaging template and on the brain surface of a glass brain rendering (bottom right) (PNG 1062 kb) [file 259_2021_5668_Fig4_ESM.png]

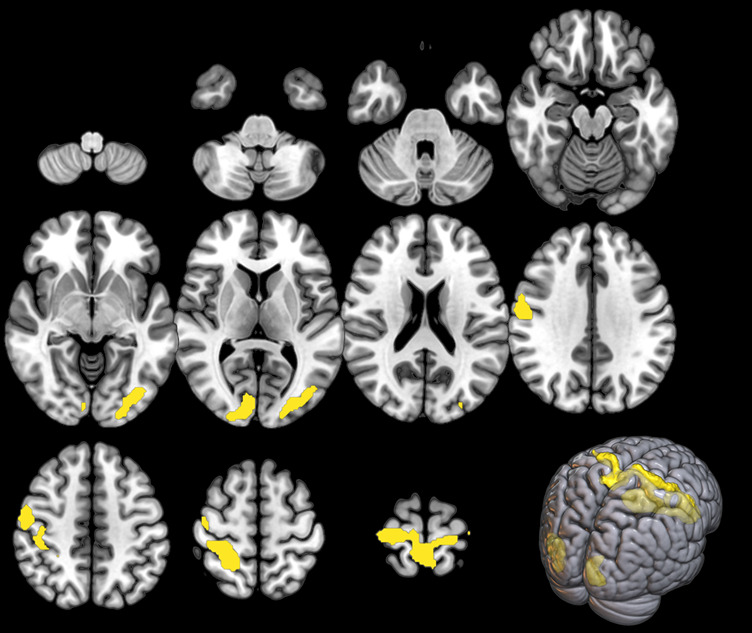

Supplement: Supplementary file 2 — High resolution (TIF 372 kb) [file 259_2021_5668_MOESM1_ESM.tif]
